# Dodecanedioic Acid: Alternative Carbon Substrate or Toxic Metabolite?

**DOI:** 10.3390/biom16010057

**Published:** 2025-12-30

**Authors:** Igor Radzikh, Usua Oyarbide, Akshay Suresh Patil, Yana I. Sandlers

**Affiliations:** 1 Department of Chemistry, Cleveland State University, Cleveland, OH 44115, USA; igorradzikh95@gmail.com (I.R.);; 2 Departments of Pediatrics and Cancer Biology, Lerner Research Institute, Cleveland Clinic, Cleveland, OH 44195, USA

**Keywords:** dodecanedioic acid, dicarboxylic acids, fatty acid oxidation, alternative carbon substrate, anaplerosis

## Abstract

Twelve-carbon dicarboxylic acid dodecanedioic acid (DODA) has gained recent interest as an alternative nutrient. However, little is known about DODA cellular metabolism. Our study presents novel data on DODA metabolism and its potential role as an alternative carbon substrate. Cells are readily oxidizing DODA as a primary carbon source, yielding acetyl-CoA and succinate and replenishing the Krebs cycle. Furthermore, cells treated with DODA are characterized by a distinct metabolic profile, whereas pathways associated with energy metabolism are highly impacted. We also found that DODA administration alters carbon substrate preferences for respiration, restricting overreliance on one substrate as a primary fuel. Consequently, by rebalancing cellular energy metabolism, DODA as a supplemental carbon source may have significant therapeutic implications in conditions that are characterized by energy deficiency and metabolic inflexibility.

## 1. Introduction

Dicarboxylic acids are endogenously produced in mammals through two primary pathways: ω-oxidation of monocarboxylic acids and β-oxidation of longer-chain dicarboxylic acids. While peroxisomes and mitochondria both participate in β-oxidation, the oxidation in peroxisomes is incomplete due to the absence of the Krebs cycle and respiratory chain complexes. The peroxisomal dicarboxylic acids oxidation intermediates are transported to mitochondria for the final oxidation and concurrent ATP production. Inherited metabolic conditions such as fatty acid oxidation disorders (i.e., medium-chain acyl-CoA dehydrogenase deficiency (MCAD deficiency), long-chain acyl-CoA dehydrogenase deficiency (LCAD deficiency), multiple acyl-CoA dehydrogenase deficiency (MADD)) are all characterized by defective mitochondrial β-oxidation [1,2,3,4]. To maintain energy balance when mitochondrial function is impaired, compensatory mechanisms such as increased peroxisomal fatty acid oxidation occur, often leading to an overproduction of dicarboxylic acids, which can be used for diagnosis [5,6].

Among dicarboxylic acids, a dodecanedioic acid (DODA), a twelve-carbon dicarboxylic acid, has been recognized as a potential nutrient [7,8] rather than a fatty acid oxidation disorders biomarker. DODA demonstrates a unique ability to generate gluconeogenic and anaplerotic substrates [9,10,11,12], although the underlying mechanisms remain to be fully elucidated. The process of replenishing Krebs cycle intermediates is particularly critical during periods of metabolic imbalance, dysregulated nutrient metabolism, or impaired mitochondrial function [13,14]. Anaplerotic therapy involves administration of an alternative carbon source to support Krebs cycle activity and ATP production. This approach has shown promise in addressing various diseases, including fatty acid oxidation disorders, propionic acidemia, pyruvate carboxylase deficiency, and cardiac and muscle diseases [13,14,15,16,17,18,19].

Besides DODA’s potential to actively replenish Krebs cycle intermediates, multiple studies have also indicated that DODA has beneficial effects on glucose regulation. Diabetic individuals who received oral DODA experienced improved exercise tolerance and reduced glucose oxidation without insulin stimulation [9,20,21]. A more recent study by Goetzman et al. also demonstrated that DODA, used as a carbon source, leads to increased metabolic rate, reduced body fat, and improved glucose tolerance in animal models [22]. These metabolic properties make DODA a promising candidate as a nutritional supplement in conditions of reduced metabolic flexibility or metabolic decompensation, particularly when certain nutrients are restricted or cannot be metabolized effectively. Despite reports highlighting DODA’s beneficial characteristics, some studies have raised concerns about its potential toxic side effects. For instance, increased hepatic triacylglycerols have been observed in mice following DODA administration [23], and prolonged fasting-induced excessive dicarboxylic acid β-oxidation can suppress mitochondrial β-oxidation, promoting hepatic lipid deposition and steatosis [24]. These findings raise some concerns regarding DODA lipotoxicity. Clinically, dicarboxylic aciduria—the urinary excretion of DCAs—is a hallmark of inherited fatty acid oxidation disorders (FAODs) [6,25]. Elevated DCA excretion reflects the activation of the ω-oxidation bypass pathway to detoxify accumulated fatty acids; thus, dicarboxylic aciduria serves as a critical biomarker correlating with severe metabolic derangements and associated toxicities, including cardiomyopathy and rhabdomyolysis. Dicarboxylic acids’ intrinsic toxicity may also be linked to unfavorable pharmacokinetic properties. Compared to analogous monocarboxylic fatty acids, DCAs exhibit markedly weaker affinity for serum albumin, resulting in a significantly higher concentration of circulating unbound species [26]. This free fraction has been shown to exert profound structural and functional damage on isolated mitochondria, suggesting a potential role in systemic mitochondrial disorders such as Reye’s syndrome [26,27].

This work aims to investigate DODA as an alternative carbon source and its wide metabolic impact on human fibroblasts. Even though fibroblasts have low metabolic activity, they are often collected through skin biopsies by clinicians as a cellular model in inherited metabolic disorders [28,29,30,31,32,33,34,35]. Many of these inherited conditions are manifested with an energy deficit through metabolic inflexibility and may benefit from alternative carbon sources such as DODA.

## 2. Materials and Methods

### 2.1. Cells

Human fibroblasts (GM00302, GM08680, and GM08389) were purchased from Coriell Institute for Medical Research, Camden, NJ, USA. Approval of the Institutional Review Board was not required upon purchase. The material transfer agreement was approved by the Cleveland State University institutional board. Cells were maintained in high-glucose DMEM medium (Corning, USA #10-013-CV) containing 10% fetal bovine serum (FBS) (ThermoFisher Scientific, USA #10437-010) and 100 mg/mL penicillin (Quality Biological, USA #120-095-721) at 37 °C at 5% CO_2_.

### 2.2. Cell Viability Assay

A total of 5000 cells/well was plated in 96-well plates and cultured for 24 h at 37 °C at 5% CO_2_. Following the incubation period, the cell culture medium was removed, and the cells in the control group were supplemented with 100 μL of DMEM glucose-free medium containing 5% FBS for 24 h. (+) DODA groups were additionally supplemented with DODA at a range of concentrations: 0.05–20 mM. After the 24 h treatment, the culture medium was aspirated, and 100 μL of a 0.5 mg/mL 3-(4,5-Dimethylthiazol-2-yl)-2,5-Diphenyltetrazolium Bromide (MTT) solution was added, followed by a 2 h incubation. Following incubation, the MTT solution was removed, and 200 µL of DMSO was added, with 20 min of shaking at room temperature. Absorbance was measured at 570 nm using a SpectraMax M2 (Molecular Devices, San Jose, CA, USA) microplate reader and analyzed with SoftMax Pro 6 (Version: 6.4). Acquired values were transferred to GraphPad Prism (Version 5.01) for statistical analysis.

### 2.3. Glucose Uptake Assay

Prior to the experiment, cells were seeded in 6-well plates. At 80% confluency, cell medium was aspirated, and cells were washed twice with 1 mL of PBS (Quality Biological, USA #114-059-101). Cells were cultured with DMEM ((-) glucose) (Gibco, USA,#11966-025) medium supplemented with 5 mM of ^13^C_6_-glucose (Sigma Aldrich, USA 389374-2.00G), 5% FBS, and 100 mg/mLpenicillin. (+) DODA cell lines were also supplemented with 3 mM of DODA (Millipore Sigma, USA D1009-100G). Cells were loaded with DODA for the course of 24 h. Every eighth hour, 50 µL aliquots of medium were aspirated for ^13^C_6-_glucose uptake measurement. ^13^C_6_-glucose in media was analyzed using a modified version of the method described by House et al. [36]. Unlabeled galactose was used as an internal standard. Galactose and ^13^C_6_-glucose peaks were resolved by gas chromatography and mass spectrometry (GCMS) [37]. For the analysis, the 10 µL aliquots of medium were transferred to a microcentrifuge tube. Samples were spiked with 10 µL galactose (100 µM) as the internal standard, vortexed for 1 min, and centrifuged at 10,000× *g* for 10 min at 4 °C. The supernatant was removed and dried under nitrogen at room temperature. The derivatization for GCMS analysis was performed with 40 µL hydroxylamine for 30 min at 80 °C, followed by 60 µL of acetic acid for another hour to yield glucose and galactose aldonitrile pentaacetate. Derivatized samples were dried under nitrogen, reconstituted in 100 µL of ethyl acetate, transferred to an autosampler, and 1 µL was injected into the EI-GC/MS. The targeted monitored ions were ^13^C_6_-glucose *m*/*z* 319 and ^12^C_6_-galactose *m*/*z* 314.

### 2.4. ^13^C_12_-DODA Uptake and Incorporation into Krebs Cycle Intermediates

Prior to the experiment, cells were seeded in 6-well plates. Upon reaching 80% confluency, the medium was aspirated, and cells were washed twice with 1 mL of PBS. Control cells were supplied with DMEM (-) glucose (Gibco, 11966-025) medium supplemented with 3 mM of ^13^C_12_-DODA (Toronto Research Chemicals, 1173019-20-9), 5% FBS, and 100 mg/mL penicillin. Cells were treated for a course of 24 h. Every 8 h, 50 µL aliquots of medium were aspirated for ^13^C_12_-DODA uptake measurement. Aliquots of 10 µL were transferred to a microcentrifuge tube. Samples were spiked with 10 µL undecanedioic acid (100 µM) as an internal standard. The pH in samples was adjusted with 6 N HCl to pH 3. An additional 100 µL of ethyl acetate was added to each sample, and samples were vortexed and centrifuged at 10,000 rpm for 10 min at 40 °C. Supernatant was removed and dried under nitrogen. Samples were derivatized using 100 µL *N*-tert-butyldimethylsilyl-*N*-methyltrifluoroacetamide (MTBSTFA) for 45 min at 70 °C.

Krebs cycle intermediates were extracted from cell pellets. Briefly, after medium aspiration, cells were washed with cold PBS and harvested with 2 mL of cold methanol/0.5 *v*/*v*% acetic acid. Cell pellets were scraped from plates, and lysates were centrifuged at 5000 rpm for 10 min. The supernatants were collected into the new tubes and dried. Samples were derivatized using 100 µL N-tert-butyldimethylsilyl-N-methyltrifluoroacetamide (MTBSTFA) for 45 min at 70 °C. Derivatized samples were transferred to an autosampler, and 1 µL was injected into the GC/MS. Targeted ions and GCMS conditions can be found in Table S1 (Supporting Information).

### 2.5. Seahorse Analysis

Oxygen consumption rate (OCR) and extracellular acidification rate (ECAR) were measured with an XF96 Extracellular Flux Analyzer (Agilent Technologies, USA) in accordance with the mito stress test kit (Agilent, 103015-100). A total of 5000 cells/well was plated in 96-well plates and cultured for 24 h in DMEM at 37 °C with 5% CO_2_. Following the incubation period, the cell culture medium was removed, and cells were supplemented with 100 μL of DMEM glucose-free medium containing 5% FBS for 24 h. Then, cells were incubated with the following substrates for five days: 5 mM glucose, 5 mM glucose + 3 mM DODA, 5 mM galactose, 5 mM galactose + 3 mM DODA, 0.2 mM palmitate, 0.2 mM palmitate + 3 mM DODA, 7 mM BHB, 7 mM BHB + 3 mM DODA. After a 5-day period, the cell media were aspirated, and cells were washed with 200 μL of 1× PBS. Mitochondrial respiration parameters were measured over 120 min in accordance with the manufacturer’s recommendation. At the 20 min mark, an additional aliquot of matching substrates was provided. Respiration-related parameters were measured after subsequent addition of 1.5 μM oligomycin, 1 μM carbonyl cyanide 4-(trifluoromethoxy) phenylhydrazone (FCCP), 1 μM antimycin A, and 1 μM rotenone.

### 2.6. Metabolomics Analysis and Lipid Profiling

Cells were seeded in 6-well plates under standard conditions (DMEM, 4.5 gr/L glucose, FBS (10%), penicillin (100 units/mL)). Once cells reached 80% confluency, the medium was aspirated, and cells were washed twice with 1 mL of PBS (Quality Biological, #114-059-101) before being supplemented with DMEM/(-)glucose and a single nutrient or combination of nutrients for the following conditions: 5 mM glucose, 5 mM galactose, 0.2 mM palmitic acid, 7 mM BHB, 3 mM DODA, 5 mM glucose + 3 mM DODA, 5 mM galactose + 3 mM DODA, 0.2 mM palmitic acid + 3 mM DODA, 7 mM BHB + 3 mM DODA. At the end of the study period, metabolism was quenched with 2 mL methanol/0.5 (*v*/*v*%) acetic acid. Liquid–liquid extraction was performed with 2:2:1 methanol/chloroform/water (*v*/*v*/*v*%) to separate polar metabolites from lipids. The upper and lower phases were dried under nitrogen and analyzed separately. The upper phase was reconstituted in 150 µL of methanol/water 1:1 (*v*/*v*%). The lower phase was reconstituted in 100 µL of 67:33 chloroform/methanol (*v*/*v*%).

Samples were analyzed using an Agilent 1290 Infinity II LC system coupled to an Agilent quadrupole time-of-flight mass spectrometer (6545 QTOF, Agilent Technologies) equipped with a dual AJS source. Chromatographic separation of polar upper phase metabolites was achieved with a Waters Atlantis Premier HILIC-Z (2.1 mm × 150 mm, 1.7 µm) column. The mobile phase for the negative acquisition mode consisted of (A) 10 mM ammonium acetate, 5 μM medronic acid in aqueous solution adjusted to pH 9 with ammonium hydroxide, and (B) 10 mM ammonium acetate, 5 μM medronic acid in acetonitrile/water (85/15 *v*/*v*%). The mobile phase for the positive acquisition mode was composed of (A) 10 mM ammonium formate, 0.1% formic acid in aqueous solution, and (B) 10 mM ammonium formate, 0.1% formic acid in water/acetonitrile (10:90 *v*/*v*%). The gradient elution profile for both ionization modes was as follows: 0–1 min, 90% B; 1–21 min, 70% B; 21–23 min, 40% B; 23–28 min, 10% B; 28–30 min, 10% B; 30–32 min, 90% B; 32–34 min, 90% B. The post-run equilibration time was set to 5 min. The flow rate was set to 0.400 mL/min. The column temperature was set to 35 °C. Injection volume was 3 µL. All samples were analyzed in the positive and negative ion modes separately. The source parameters were set as follows: drying gas (N_2_) temperature 300 °C and flow 11 L/min, sheath gas (N_2_) temperature 350 °C and flow 11 L/min; nebulizer gas (N_2_) pressure 40 psi; capillary voltage 2000/3000 (−/+); nozzle voltage 0 V; fragmentor voltage, 70 V; skimmer voltage, 30 V; and octupole radiofrequency voltage (OCT RF V), 750 V. Collision energies (CE) for the MS/MS were set to 0, 15, 30, and 45 eV. The scan range was set from 60 to 1700 *m*/*z* with a scan rate of 1 spectra/s for MS. The MS/MS scan range was set from 60 to 1700 *m*/*z* with a scan rate of 5 spectra/s. In the positive mode, reference masses were 121.0509 and 1221.990637, and in the negative mode, 119.03632 and 980.016375.

Data was processed with Agilent MassHunter Profinder software (Version: B.10.0.2) for batch recursive feature extraction analysis. Prior to molecular feature extraction, retention time alignment was performed using an internal standard (Succinate-d4, #293075, Sigma Aldrich). Peak areas were normalized to total cellular protein (BCA protein kit/Thermo Scientific #J63283QA). Feature identification was performed using an in-house built MS/MS library combined with METLIN AM (Accurate Mass). Unidentified metabolites were subject to molecular formula generation. Data were then exported to Metaboanalyst 6.0 for statistical and pathway analysis.

Chromatographic separation of lipids was achieved with a T3 HSS column (2.1 mm × 100 mm, 2.1 µm) Waters (Milford, MA, USA). The mobile phase for the negative acquisition mode was (A) 0.1% acetic acid in 60:40 water/acetonitrile (*v*/*v*%) and (B) 90:10 isopropanol/acetonitrile (*v*/*v*%), 0.1% acetic acid. The mobile phase for the positive acquisition mode was as follows: (A) 0.1% formic acid in 60:40 water/acetonitrile (*v*/*v*%) and (B) 90:10 isopropanol/acetonitrile (*v*/*v*%), 0.1% formic acid. The gradient elution profile for both modes was 0–1 min, 60% B; 1–30 min, 90% B; 30–31 min, 90% B; 31–32 min, 40% B; 32–33 min, 60% B. The post-run equilibration time was set to 3 min. The flow rate was set at 0.200 mL/min. The column temperature was 45 °C. The injection volume was 5 µL. Iterative MS/MS acquisition (four consecutive injections) was performed with a pooled QC sample for generation of an in-house build library utilizing Lipid Anotator (Version B. 1.0.54).

### 2.7. Statistical Analysis

Results are expressed as the mean ± standard errors of the mean (*n* = 6 for the Seahorse experiment and *n* = 3 for the rest of the studies). Statistical significance was set at *p* < 0.05, two-tailed unpaired *t*-test. Multivariate statistical analysis for metabolomics data was performed with Metaboanalyst 6.0.

## 3. Results

### 3.1. Establishing Dose-Dependent Curves

**Figure 1:**
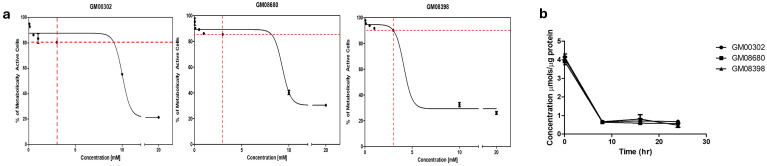
(**a**) Dose–response curves by cellular metabolic activity/cell viability MTT assay in the presence of DODA at 0.05–20 mM for three cell lines; (**b**) DODA uptake from cellular media.

Due to concerns about the potential toxicity of dicarboxylic acids, we conducted a dose–response study of DODA’s toxicity using the 3-(4,5-dimethylthiazol-2-yl)-2,5-diphenyltetrazolium bromide (MTT) cell viability assay. The range of 0.05–20 mM DODA concentrations was tested in three fibroblast cell lines collected from healthy individuals (GM00302, GM08680, GM008398; (Figure 1a). During this experiment, all cells were depleted of major carbon sources and supplemented with DODA. All cell lines showed a similar trend: a moderate decrease in cellular metabolic activity at the 3 mM DODA level, preceding a significant reduction in overall metabolic activity. Since eighty percent of cells were still metabolically active at 3 mM, this concentration was chosen for all future experiments.

### 3.2. DODA Is Consumed by Fibroblasts and Its Carbons Refill Krebs Cycle

Human studies reveal that DODA’s pharmacokinetic profile is characterized by rapid absorption, efficient tissue utilization, and minimal urinary excretion [10]. To explore its cellular uptake, we supplemented cell growth media with 3 mM DODA. We then monitored the exogenously added DODA levels in the media over 24 h (Figure 1b). Remarkably, after just 10 h, all three cell lines showed a significant decrease in medium DODA, with roughly 85% being consumed.

Several studies suggest that DODA has anaplerotic properties [12,38] because the end products of DODA’s oxidative metabolism, succinate and acetyl-CoA, can enter the Krebs cycle. Thus, we used ^13^C-labeled DODA (^13^C_12_DODA) to monitor incorporation of ^13^C at the entry points to the Krebs cycle. During chromatographic separation, retention times of ^13^C-labeled metabolites remain the same as unlabeled; however, the ^13^C isotope has a nominal mass of 13.00335 Da, and ^13^C-labeled metabolites can be resolved by mass spectrometer from ^12^C-unlabeled molecules. Hereafter, the unlabeled metabolites will be referred to as M0. Metabolites with n number of ^13^C-labeled carbons will be referred to as Mn.

Analysis of ^13^C enrichment in Krebs cycle intermediates revealed distinct labeling patterns in citrate, succinate, fumarate, and malate. Citrate primarily exhibited M2 and M6 isotopomers, consistent with incorporation of ^13^C from M2 acetyl-CoA and the combination of M2 acetyl-CoA with M4 oxaloacetate, respectively. M4 succinate can theoretically arise from M2 acetyl-CoA after three or more complete turns of the Krebs cycle; however, this requires extensive isotopic scrambling and assumes production of M4 citrate, which we found negligible (at the limit of quantification).

M2 succinate can be produced directly from M2 acetyl-CoA or from M4 succinate after several Krebs cycle turns. In the latter case, M4 citrate is also expected to be produced.

**Figure 2:**
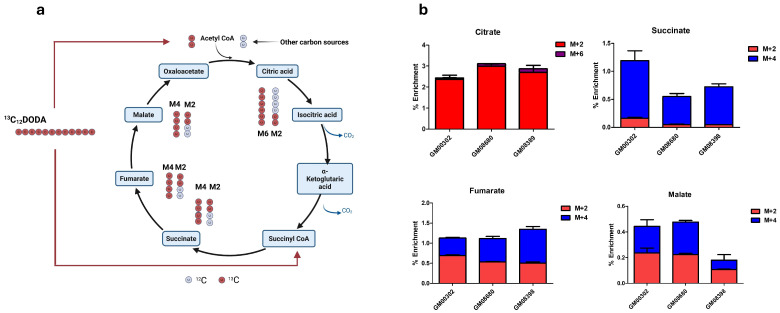
(**a**) ^13^C fate from ^13^C_12_DODA into Krebs cycle intermediates. For simplicity, only isotopomers’ patterns from the first Krebs cycle turn are shown. See explanation in the text. Unlabeled carbons are from other nutritional sources (for example, branch chain amino acids or glutamine). (**b**) Percent enrichment in detected Krebs cycle intermediates.

While all the above-mentioned scenarios are possible, we consider direct entry of M4 succinate from ^13^C_12_ DODA oxidation to be the more plausible explanation (Figure 2a). DODA is known to induce peroxisomal enzyme expression [39], and in liver tissue, it undergoes peroxisomal β-oxidation to yield succinate [12], which then enters the mitochondrial Krebs cycle. This mechanism aligns with our observation of significant M4 enrichment in succinate, fumarate, and malate after incubation with ^13^C_12_DODA, supporting the hypothesis that peroxisomal oxidation of DODA contributes directly to the succinate pool [38].

### 3.3. DODA Decreases Cellular Uptake of Glucose

**Figure 3:**
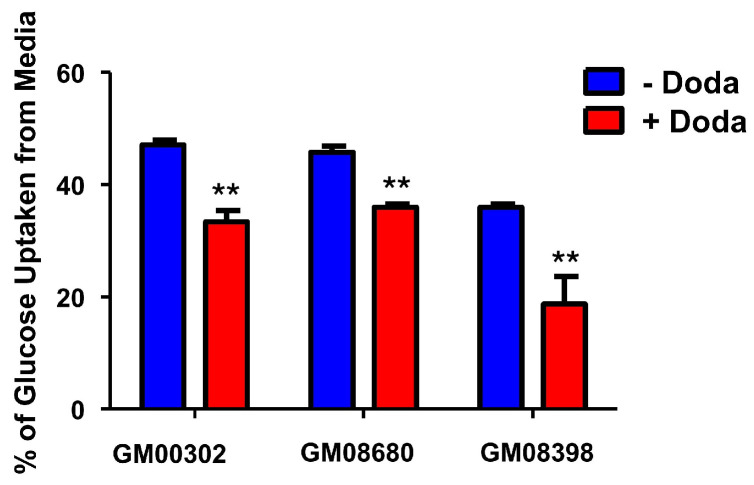
^13^C_6_-Glucose uptake from the cellular media. ** *p* < 0.05.

As an exogenously supplemented carbon source, DODA competes with other essential nutrients. Human studies have shown a significant decrease in whole-body glucose uptake during DODA infusion [20]. Similarly, rodent models have demonstrated that DODA administration impacts glucose kinetics, affecting both uptake and utilization rates [9]. We evaluated glucose uptake in the presence of DODA by the disappearance of labeled ^13^C_6_-glucose from the cellular media over time (every eight hours). All cell lines treated with 3 mM DODA exhibit decreased ^13^C_6_-glucose cellular uptake (Figure 3).

### 3.4. Changes in Metabolic and Lipid Profiles

**Figure 4:**
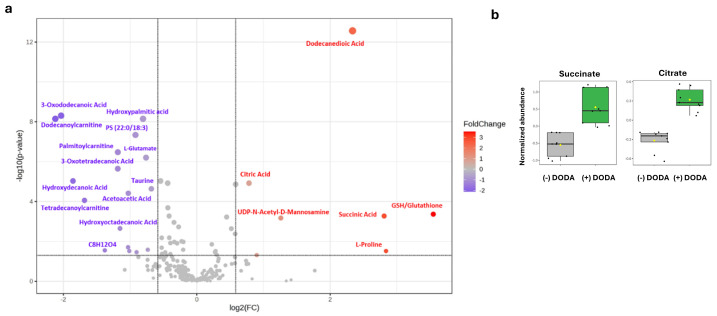
(**a**) Volcano plot shows statistically significant altered metabolites (*p* < 0.05 and fold of change FC > 1.5); (**b**) succinate and citrate levels in control (−) DODA and (+) DODA cells.

Untargeted metabolomics analysis of (−) DODA cells and (+) 3 mM DODA for 36 h was performed. To reduce experimental variability, all cell lines were grown under the same conditions and were harvested simultaneously using the same batch of solvents and internal standards. A hydrophilic interaction liquid chromatography–high resolution mass spectrometry (HILIC-HRMS) method was employed to analyze polar metabolites. For the untargeted metabolomics statistical analysis, we created two experimental groups: (-) DODA and (+) DODA. Each group consisted of three different fibroblast lines tested in triplicate. Peak areas were normalized to the internal standard (Succinate-d_4_) and total cellular protein, followed by *log* transformation and Pareto scaling. The generated volcano plot (Figure 4a) demonstrates that 3 mM DODA administration leads to seventeen significantly decreased and seven significantly increased metabolites. Among the elevated metabolites were succinate and citrate (Figure 4b), both crucial entry points to the Krebs cycle. The altered metabolites also encompassed amino acids, reduced glutathione (GSH), hydroxy-fatty acids, and various long-chain acylcarnitines.

The accumulation of long-chain acylcarnitines (LCACs) serves as a key clinical biomarker for fatty acid oxidation disorders and is also observed in conditions like ischemic myocardium and heart failure [40,41,42,43]. Elevated levels of long-chain acylcarnitine are detrimental to mitochondrial function [40] and cardiac electrophysiology [44]. Emerging evidence underscores the potential of LCACs as therapeutic targets to mitigate ischemia-reperfusion injury [45,46]. Our recent work established that 1 mM DODA significantly reduces various long-chain acylcarnitines in very long-chain acyl-coenzyme A dehydrogenase (VLCAD)-deficient fibroblasts [47]. The present untargeted metabolomics analysis provides further support and expands upon these targeted findings, laying the groundwork for future investigations into the impact of DODA and other dicarboxylic acids on cardiac electrophysiological derangements.

Significantly increased glutathione (GSH) is another metabolite identified by an untargeted metabolomics study (Figure 4a). Glutathione, a powerful cellular antioxidant, is synthesized from glutamate, cysteine, and glycine. During reaction with reactive oxygen species (ROS), reduced glutathione is converted to oxidized glutathione (GSSG) by glutathione peroxidase, and to make a meaningful conclusion, both GSH and GSSG are required. In our study, only GSH was identified with high confidence (confirmed with retention time and tandem mass spectrometry fragmentation patterns of GSH standard). The GSH level is significantly increased, along with the finding that one of the GSH precursors, glutamate, is depleted. Glutamate is involved in various metabolic pathways, playing an essential role in multiple processes. In the context of our data, glutamate links proline synthesis and carbon flux to the Krebs cycle and glutathione synthesis. Since DODA carbons are in competition with other carbon substrates to enter the Krebs cycle, we speculate that there is a possible up-regulation of GSH and proline production as an adaptive mechanism to channel glutamate to alternative pathways, away from feeding into the Krebs cycle. This hypothesis will need to be further investigated in detail with appropriate stable isotope tracers.

**Figure 5:**
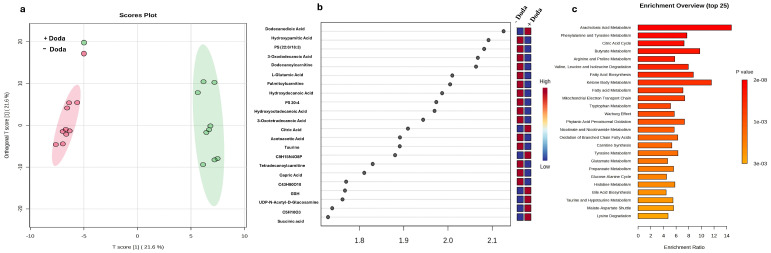
(**a**) OPLS-DA shows separation in metabolome of control cells (−) DODA and DODA-treated cells (+DODA). The shaded areas indicate the 95% confidence intervals based on the data points for individual groups; (**b**) variable importance in projection (VIP) scores for the top metabolites contributing to variation in metabolic profiles of controls vs. DODA-treated. The relative abundance of metabolites is indicated by a colored scale from blue to red, representing low and high, respectively; (**c**) metabolic set enrichment analysis demonstrates the most enriched pathways.

We further used orthogonal partial least square discriminate analysis (OPLS-DA) to get better insight into the distinct metabolic profile of cells treated with DODA. OPLS-DA resulted in the visual separation of metabolic profiles in multidimensional space, demonstrating a distinct clustering of control (−) DODA vs. treated (+) DODA cells (Figure 5a). The supervised OPLS-DA model yielded a satisfactory fitness of the model and predictive ability values (R^2^ = 0.867 and Q^2^ = 0.796). Variable importance in the projection (VIP) scores were analyzed (cut-off VIP > 1) to rank identified metabolites according to their contribution to the capability to discriminate metabolic profiles (full list of metabolites can be found in Supporting Information (Table S2)). The top twenty-two metabolites of interest are shown separately (Figure 5b). Most of the identified metabolites with the highest VIP scores represent metabolites that are involved in fatty acid oxidation and Krebs cycle pathways.

We selected the identified metabolites to perform a pathway enrichment analysis using the Kyoto Encyclopedia of Genes and Genomes (KEGG) database. This analysis effectively revealed key pathways affected by the DODA treatment. Notably, multiple pathways related to amino acid and fatty acid metabolism, as well as the Krebs cycle and mitochondrial electron transport chain, showed a high enrichment fold and a statistically significant *p*-value (Figure 5c).

**Figure 6:**
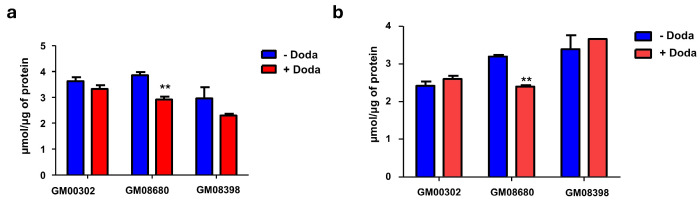
Total triglyceride (**a**) and diglyceride (**b**) levels. ** *p* < 0.05.

We then further examined the distribution of different lipid classes in both the (−) DODA and (+) DODA cells. Our analysis showed that the total number of detected and identified lipids per lipid class did not change after DODA treatment (Supporting Information, Table S3). Additionally, we found no accumulation of total triglycerides and diglycerides (Figure 6).

### 3.5. DODA Impact on Mitochondrial Bioenergetics

To gain insight into DODA as an alternative carbon substrate, we have studied mitochondrial respiration through the mitochondrial stress assay (Seahorse XFe96 Analyzer/Agilent). Fibroblasts (GM08680) were supplemented with various carbon sources. We used either a single primary substrate—glucose (5 mM), galactose (5 mM), palmitate (0.2 mM), or β-hydroxybutyrate (BHB, 7 mM)—or a combination of one of these with 3 mM DODA. Additionally, a control condition was included where 3 mM DODA was administered as the sole carbon source ((-) DODA). Cells were first preincubated with specific nutrients for five days. This was followed by an acute treatment, where matching carbon sources were administered again at the 20 min mark. Real-time oxygen consumption rate (OCR) measurements provide a direct assessment of mitochondrial respiratory capacity and, consequently, the cell’s energetic state.

The five-day pre-incubation period for this experiment was intentionally chosen to model chronic substrate exposure rather than an acute intervention. This design reflects the pathophysiological condition of metabolic inflexibility and/or possible therapeutic context where alternative carbon sources, such as dodecanedioic acid (DODA), would be administered over extended periods of time. Chronic exposure allows cells to reach metabolic equilibrium and engage in adaptive processes, including enzyme-level regulation and remodeling of mitochondrial bioenergetics, which cannot be captured in short-term assays. While prolonged treatment may influence the whole-body glycogen or lipid stores, these changes represent the integrated metabolic phenotype induced by sustained substrate utilization.

**Figure 7:**
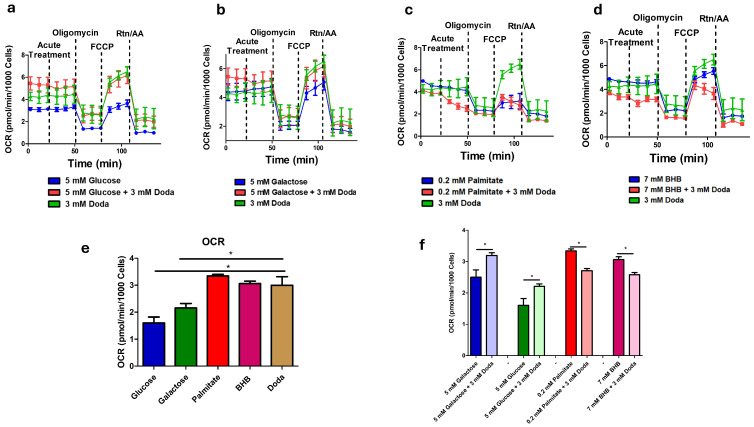
(**a**–**d**) Oxygen consumption rate profiles. (**e**,**f**) Total oxygen consumption rates (120 min) under different substrates. Data presented as mean (*n* = 6) ± SEM. * *p* < 0.05.

Theoretically, the oxidation of a single DODA molecule demands between 13.5 and 15.5 oxygen molecules [22]. This oxygen requirement is greater than that for glucose oxidation yet less than that for palmitate [48]. Indeed, when DODA serves as the primary carbon source, cells exhibit a higher overall oxygen consumption rate (OCR) compared to the OCR in the presence of glucose (Figure 7a) or galactose (Figure 7b). This suggests that mitochondrial respiration is the predominant process consuming oxygen during DODA’s metabolism. Addition of DODA to carbohydrate-based media further increases OCR; however, adding it to palmitate or β-hydroxybutyrate (BHB) leads to a decrease in overall OCR (Figure 7c and Figure 7d, respectively). Acute exposure (at 20 min mark) to the combination of palmitate + DODA (Figure 7c) resulted in a significant decrease in the oxygen consumption rate. This reduction in OCR likely stems from the elevated NADH/NAD+ ratio caused by immediate DODA oxidation. The increased NADH/NAD+ ratio inhibits palmitate oxidation, a conclusion further supported by metabolomic analysis, which revealed a concomitant decrease in palmitoyl carnitine levels (Figure 4a/volcano plot), an intermediate in palmitate oxidation.

The analysis of overall oxygen consumption rates (OCRs) with various carbon sources (Figure 7e,f) shows that DODA significantly boosts cellular respiration when carbohydrates are present. However, the mechanism behind the observed decrease in OCR when DODA is combined with β-hydroxybutyrate (BHB) is yet to be elucidated. It is important to note that the 7 mM BHB concentration employed here represents a state of severe ketosis, similar to that found in diabetic ketoacidosis or extreme nutritional ketosis, rather than the physiological ketosis of fasting. Consequently, future investigations should focus on exploring the effects of lower, more physiologically relevant BHB concentrations on cellular respiratory activity.

**Figure 8:**
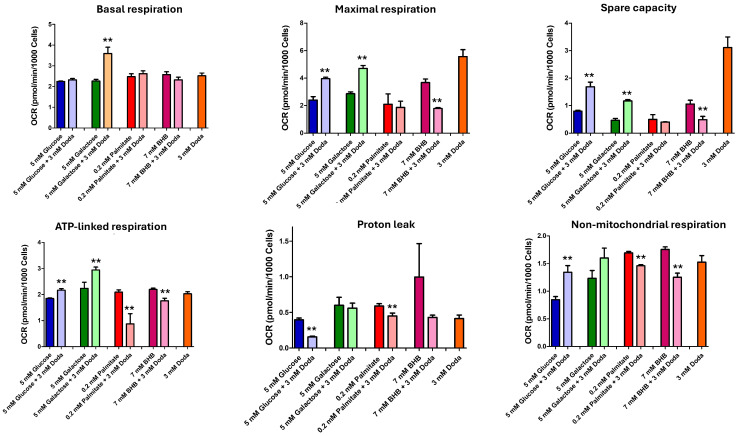
Calculated cellular respiration characteristics. Data presented as mean (*n* = 6) ± SEM for each condition. ** *p* < 0.05.

Calculated basal respiration rates (Figure 8) reflect the cellular energetic status under the five-day baseline treatment conditions. This metric represents the sum of all oxygen-consuming cellular processes, including oxidases and peroxisomal activities. Notably, cells cultured with DODA as the primary carbon source showed basal respiration rates comparable to those observed with other substrates. We also calculated the reserve capacity after FCCP addition (Figure 8), which acts as an uncoupler in this instance. Reserve capacity is a function of both basal and maximal respiration rates. Both the maximal respiration rate and spare respiratory capacity were significantly higher with DODA as a nutrient compared to other substrates (Figure 8). While the maximal respiration rate is mainly determined by carbon source availability and oxidation, reserve capacity is thought to reflect a cell’s metabolic adaptability to increased ATP demand and its stress tolerance. This means that 3 mM DODA supplementation improves a cell’s ability to respond to increased energy demands. Next, we calculated ATP-linked respiration (Figure 8) that is governed by ATP utilization (energy demand), ATP synthesis, and carbon substrate supply and oxidation. In cells treated with DODA for five days, ATP-linked respiration did not surpass the ATP yield observed with palmitate or β-hydroxybutyrate (BHB). The amount of ATP generated per DODA molecule is contingent upon the degree of peroxisomal oxidation. Tissues rich in peroxisomes (e.g., liver, kidney) are predicted to yield less ATP from DODA than those with lower peroxisome abundance (e.g., muscle, heart) [22]. We found that ATP-linked respiration significantly increased when DODA was given alongside glucose or galactose. In contrast, combining DODA with palmitate or β-hydroxybutyrate (BHB) led to a notable decrease in ATP-linked respiration compared to using palmitate or BHB alone as carbon sources.

**Figure 9:**
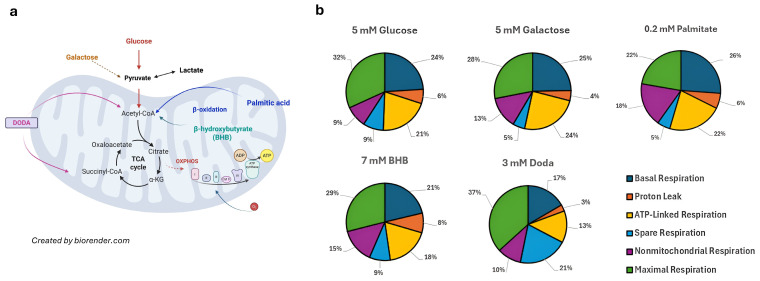
(**a**) Carbon substrates under the current study. Both glucose and fatty acids contribute to the same mitochondrial acetyl-CoA pool, but the relative contribution shifts depending on the cell’s energy state. Oxygen consumption rate (**b**) partitioning for each substrate. In this analysis, total OCR was established as one hundred percent.

The analysis of oxygen consumption rate partitioning for each substrate (Figure 9a) further demonstrates that DODA as a primary carbon source has a major impact on spare capacity and maximal respiration (total OCR was established as 100%, Figure 9b).

We also measured extracellular acidification rate (ECAR) concurrently with oxygen consumption rate (OCR). ECAR reflects medium pH changes and primarily indicates glycolytic flux when oxidative phosphorylation (OXPHOS) is limited, representing anaerobic glycolysis or, in glucose-free conditions, pyruvate-to-lactate conversion. During these processes, proton extrusion acidifies the medium, and the change in pH is measured. Although Krebs cycle-derived CO_2_ also contributes to acidification, anaerobic glycolysis is the dominant factor.

**Figure 10:**
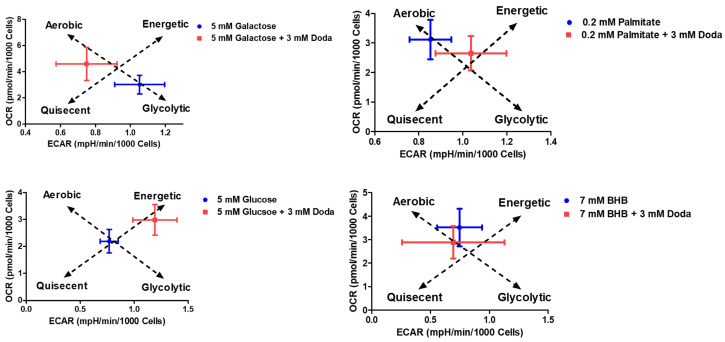
OCR vs. ECAR profiles under different substrates.

Analysis of OCR and ECAR profiles over 150 min (Figure 10) revealed substrate-dependent shifts in cellular metabolism, particularly when glucose/galactose were combined with DODA. The observed shift towards a more energetic phenotype in cells treated with glucose + DODA compared to glucose alone is likely attributed to the preferential oxidation of DODA in the presence of glucose. This is supported by the sharp decrease in ECAR following acute (20 min mark) glucose + DODA administration (Figure S1, Supporting Information), suggesting that acute DODA treatment promotes a shift from glycolytic flux toward a more aerobic state characterized by reduced pyruvate-to-lactate conversion.

## 4. Discussion

Many cells possess remarkable metabolic flexibility, utilizing a variety of carbon substrates to fuel their bioenergetic needs. Substrate preferences vary, depending on physiological conditions, circulating nutrient availability, and disease states. The interplay between glucose oxidative metabolism and the β-oxidation of monocarboxylic fatty acids for efficient ATP production is a metabolic determinant of proper cellular function, especially in the heart and muscle. When the balance between fatty acid and glucose oxidative metabolism is disrupted, it leads to what is known as metabolic inflexibility—a phenomenon now targeted as a therapeutic approach for numerous heart diseases. A maladaptive cellular response to metabolic inflexibility often occurs when oxygen demand surpasses supply, shifting metabolism towards anaerobic glycolysis and producing lactate. This shift is characteristic of intense exercise, ischemia, chronic heart failure, and hypertrophic cardiomyopathy. A maladaptive over-reliance on glucose as a primary carbon source nutrient (and as a result, metabolic lactic acidosis and hypoglycemia) is a hallmark of some inherited metabolic disorders with various degrees of OXPHOS dysfunction, or reduced capacity for fatty acid oxidation. Fatty acid oxidation disorders [49], Barth syndrome, and mitochondrial disorders [50] all present with ATP deficiency and lactic acidosis [6,51,52]. Lactate accumulation may induce uncoupling of glycolysis from glucose oxidation, exacerbating energy production constraints in these pathologies [53]. There is also an association between elevated cardiac lactate and arrhythmias, potentially due to the low pH effect on myocardial electrophysiological processes [54]. Moreover, excessive lactate production in the heart can acidify the cellular environment, reducing calcium sensitivity and impairing cardiac contractility [55].

Given these challenges in metabolic balance and the detrimental effects of conditions like metabolic lactic acidosis, dodecanedioic acid (DODA) may serve as a promising alternative carbon substrate with potential metabolic benefits. However, DODA’s comprehensive metabolic impact and any potential adverse effects are still largely unknown. Therefore, this study aims to thoroughly investigate the broad metabolic effects of DODA in human fibroblasts from healthy individuals, comparing cellular respiration parameters when using glucose, galactose, β-hydroxybutyrate, and palmitic acid as primary carbon sources, both alone and in combination with DODA. Our comprehensive investigation provides novel and critical insights into the metabolic fate and impact of dodecanedioic acid (DODA) as a versatile alternative carbon source in human fibroblasts.

DODA is readily consumed and oxidized by cells, actively replenishing the Krebs cycle primarily through acetyl-CoA and succinate. This carbon flux characteristic is particularly significant, as it directly supports cellular energy metabolism, a crucial aspect in conditions marked by Krebs cycle dysfunction and energy deficit. We observed that DODA administration leads to a distinct metabolic profile, and pathways associated with energy metabolism are highly impacted. Furthermore, our bioenergetic analyses reveal DODA’s profound impact on cellular respiration. When co-administered with carbohydrates (glucose or galactose), DODA significantly enhances overall oxygen consumption rates and ATP-linked respiration, leading to an improved mitochondrial bioenergetic profile, including decreased proton leak, elevated maximal respiration, and increased spare respiratory capacity. This demonstrates DODA’s capacity to reinforce cellular resilience and adaptability to increased energy demands. We also found that cells acutely exposed to DODA exhibit a marked decrease in extracellular acidification rate (ECAR). The reduction in ECAR is attributed to the reduced glycolytic flux. In light of decreased glucose uptake, these results suggest that DODA is preferentially oxidized in the presence of carbohydrates. These characteristics suggest DODA’s potential to prevent over-reliance on glucose and mitigate risks associated with metabolic lactic acidosis and hypoglycemia.

DODA’s interaction with fatty acids is more nuanced, suggesting a potential rebalancing of substrate oxidation that warrants further investigation. Cells treated with a palmitate + DODA combination at the 20 min mark exhibited a significant drop in oxygen consumption rates, most likely due to the increase in the NADH/NAD+ ratio inhibiting palmitate oxidation. A decrease in palmitoyl carnitine, an intermediate of palmitate oxidation, further supports that palmitate oxidation is attenuated. We also found that DODA treatment leads to a decrease in toxic long-chain acylcarnitine and 3-hydroxy long-chain fatty acids. The accumulation of long-chain acylcarnitine is detrimental to mitochondrial function and is associated with an adverse clinical outcome in heart failure, whereas 3-hydroxy fatty acids, which are also intermediates of fatty acid oxidation, act as a strong uncoupler of oxidative phosphorylation, potentially altering energy homeostasis [56,57]. Notably, fatty acid oxidation inhibition is a therapeutic strategy for ischemic heart disease and heart failure [58,59,60]; however, this type of inhibition carries the risk of hypoglycemia [61]. Metabolic inflexibility, characterized by an imbalance in the utilization of fatty acids and glucose for ATP production, is a critical factor in this context. We have demonstrated that DODA provides an additional source for ATP production, impacting both palmitate and glucose metabolism; thus, our findings have a significant implication for future studies in heart failure and ischemic heart disease fields.

Recent studies have yielded conflicting data regarding the impact of DODA on lipid profiles [22,23,24]. We found that cells treated with DODA did not exhibit significant changes in the relative proportions of some lipid classes, including triglycerides. However, it is possible that individual lipid profiles may differ after a more detailed analysis.

Ketones are an additional carbon source that contributes to ATP production, predominantly during fasting. Nutritional ketosis is beneficial to physical performance [62,63], and the ketogenic diet is often used as a treatment for neurodegenerative diseases and diabetes [64,65]. In comparison to β-hydroxybutyrate (BHB), DODA exhibited comparable basal and ATP-linked respiration rates but notably outperformed BHB in terms of maximal respiration and spare capacity, while also reducing proton leak. These characteristics have an important implication: BHB oxidation is an important source of ATP in the failing heart; however, it does not improve cardiac efficiency [66,67,68,69]. Ho et al. proposed a high proton leak and ATP production uncoupling as a possible mechanism attributable to the decrease in cardiac efficiency in the heart perfused with BHB or under a ketogenic diet [66,68,70]. It is thought that DODA is oxidized in the heart primarily through mitochondrial oxidation, which yields 60 ATP molecules [22], and thus it can potentially serve as an alternative fuel under conditions of metabolic inflexibility in the heart. More studies are needed to investigate DODA metabolism in this context.

## 5. Conclusions

In summary, our findings, supported by isotopic tracing, reveal that DODA plays a highly effective role in maintaining Krebs cycle homeostasis. DODA oxidation generates two distinct Krebs cycle entry molecules: acetyl-CoA and succinate (through succinyl-CoA). DODA is a potent modulator of cellular energy metabolism, capable of rebalancing carbon substrate utilization, enhancing mitochondrial respiratory capacity, and mitigating harmful metabolites. These characteristics position DODA as a promising supplementary energy source for therapeutic strategies targeting metabolic inflexibility, mitochondrial dysfunction, and energy deficiency in various pathological states.

Future studies should employ stable isotope tracers such as ^13^C-glucose and ^13^C-palmitate to confirm the metabolic shifts suggested by our data. Our data demonstrated reduced glycolytic flux and changes in acylcarnitine profiles, indicating altered substrate preference. ^13^C-tracer-based approaches would allow precise quantification of flux through glucose oxidation and β-oxidation pathways in the presence of DODA, providing definitive evidence of its role in rebalancing carbon substrate utilization.

We also recognize that our fibroblast model exhibits a less dynamic metabolic profile compared to highly active tissues such as liver or cardiac cells, meaning certain metabolic adjustments may require more time to manifest. This behavior can vary across different in vitro systems; however, in our study, the observed response addresses the core biological question of how prolonged DODA exposure influences the metabolic profile and certain mitochondrial functions. Overall, we believe the collected data primarily reflect chronic pre-incubation effects rather than immediate changes.

Notably, at a concentration of 3 mM, the current study showed no toxic metabolic effects in our cellular model. However, the DODA metabolic impact warrants careful consideration, as dicarboxylic acids have been associated with lipid perturbations and potential toxicity under certain conditions. This balance between therapeutic potential and metabolic risk underscores the importance of further investigation into DODA’s role in health and disease.

## Data Availability

The original contributions presented in this study are included in the article/Supplementary Materials. Further inquiries can be directed to the corresponding author.

## Supplementary Materials

The following supporting information can be downloaded at: https://www.mdpi.com/article/10.3390/biom16010057/s1, Figure S1: Extracellular Acidification Rate (ECAR) profiles under different substrates; Table S1 Krebs cycle intermediates target CGMS ions; Table S2: VIP full list of metabolites; Table S3: Lipid classes detected; Table S4: Detected features untargeted analysis.

## Abbreviations

The following abbreviations are used in this manuscript:

DODA	Dodecanedioic acid
GCMS	Gas Chromatography Mass Spectrometry
HILIC	Hydrophilic Interaction Liquid Chromatography
MCAD	Medium-chain acyl-CoA dehydrogenase deficiency
LCAD	Long-chain acyl-CoA dehydrogenase deficiency
MADD	Multiple acyl-CoA dehydrogenase deficiency
DMEM	Dulbecco’s Modified Eagle Medium
FBS	Fetal Bovine Serum
MTT	3-(4,5-Dimethylthiazol-2-yl)-2,5-Diphenyltetrazolium Bromide
DMSO	Dimethyl Sulfoxide
PBS	Phosphate Buffered Saline
MTBSTFA	N-tert-butyldimethylsilyl-N-methyltrifluoroacetamide
OCR	Oxygen Consumption Rate
ECAR	Extracellular Acidification Rate
FCCP	Carbonyl cyanide 4-(trifluoromethoxy) phenylhydrazone
BHB	β-Hydroxybutyrate
VIP	Variable Importance in Projection
OPLS-DA	Orthogonal Partial Least Squares Discriminant Analysis
LCACs	Long-chain Acylcarnitines
GSH	Reduced Glutathione
GSSG	Oxidized Glutathione
ATP	Adenosine Triphosphate
NADH/NAD^+^	Nicotinamide Adenine Dinucleotide (reduced/oxidized forms)
OXPHOS	Oxidative Phosphorylation
KEGG	Kyoto Encyclopedia of Genes and Genomes

## References

[1] Tserng K.Y., Jin S.J., Kerr D.S., Hoppel C.L. (1991). Urinary 3-Hydroxydicarboxylic Acids in Pathophysiology of Metabolic Disorders with Dicarboxylic Aciduria. Metabolism.

[2] Tserng K.Y., Jin S.J., Kerr D.S., Hoppel C.L. (1990). Abnormal Urinary Excretion of Unsaturated Dicarboxylic Acids in Patients with Medium-Chain Acyl-CoA Dehydrogenase Deficiency. J. Lipid Res..

[3] Duran M., De Klerk J.B.C., Wadman S.K., Bruinvis L., Ketting D. (1984). The Differential Diagnosis of Dicarboxylic Aciduria. J. Inherit. Metab. Dis..

[4] Wajner M., Amaral A.U. (2016). Mitochondrial Dysfunction in Fatty Acid Oxidation Disorders: Insights from Human and Animal Studies. Biosci. Rep..

[5] Ranea-Robles P., Houten S.M. (2023). The Biochemistry and Physiology of Long-Chain Dicarboxylic Acid Metabolism. Biochem. J..

[6] Ruiz-Sala P., Peña-Quintana L. (2021). Biochemical Markers for the Diagnosis of Mitochondrial Fatty Acid Oxidation Diseases. J. Clin. Med..

[7] Grego A.V., Mingrone G. (1995). Dicarboxylic Acids, an Alternate Fuel Substrate in Parenteral Nutrition: An Update. Clin. Nutr..

[8] Angelini G., Russo S., Carli F., Infelise P., Panunzi S., Bertuzzi A., Caristo M.E., Lembo E., Calce R., Bornstein S.R. (2024). Dodecanedioic Acid Prevents and Reverses Metabolic-Associated Liver Disease and Obesity and Ameliorates Liver Fibrosis in a Rodent Model of Diet-Induced Obesity. FASEB J..

[9] Bertuzzi A., Mingrone G., De Gaetano A., Gandolfi A., Greco A.V., Salinari S. (1997). Kinetics of Dodecanedioic Acid and Effect of Its Administration on Glucose Kinetics in Rats. Br. J. Nutr..

[10] Mingrone G., Greco A., De Gaetano A., Tataranni A., Raguso C., Castagneto M. (1994). Pharmacokinetic Profile of Dodecanedioic Acid, a Proposed Alternative Fuel Substrate. J. Parenter. Enter. Nutr..

[11] Mingrone G., Castagneto M. (2006). Medium-Chain, Even-Numbered Dicarboxylic Acids as Novel Energy Substrates: An Update. Nutr. Rev..

[12] Tserngg K.-Y., Jin S.-J. (1991). Metabolic Conversion of Dicarboxylic Acids to Succinate in Rat Liver Homogenates. A Stable Isotope Tracer Study. J. Biol. Chem..

[13] Owen O.E., Kalhan S.C., Hanson R.W. (2002). The Key Role of Anaplerosis and Cataplerosis for Citric Acid Cycle Function. J. Biol. Chem..

[14] Gibala M.J., Young M.E., Taegtmeyer H. (2000). Anaplerosis of the Citric Acid Cycle: Role in Energy Metabolism of Heart and Skeletal Muscle. Acta Physiol. Scand..

[15] Roe C.R., Mochel F. (2006). Anaplerotic Diet Therapy in Inherited Metabolic Disease: Therapeutic Potential. J. Inherit. Metab. Dis..

[16] Mallet R.T., Olivencia-Yurvati A.H., Bünger R. (2018). Pyruvate Enhancement of Cardiac Performance: Cellular Mechanisms and Clinical Application. Exp. Biol. Med..

[17] Longo N., Price L.B., Gappmaier E., Cantor N.L., Ernst S.L., Bailey C., Pasquali M. (2017). Anaplerotic Therapy in Propionic Acidemia. Mol. Genet. Metab..

[18] Roe C.R., Brunengraber H. (2015). Anaplerotic Treatment of Long-Chain Fat Oxidation Disorders with Triheptanoin: Review of 15 Years Experience. Mol. Genet. Metab..

[19] Wehbe Z., Tucci S. (2019). Therapeutic Potential of Triheptanoin in Metabolic and Neurodegenerative Diseases. J. Inherit. Metab. Dis..

[20] Greco A.V., Mingrone G., Capristo E., Benedetti G., De Gaetano A., Gasbarrini G. (1998). The Metabolic Effect of Dodecanedioic Acid Infusion in Non-Insulin-Dependent Diabetic Patients. Nutrition.

[21] Mingrone G., Castagneto-Gissey L., Macé K. (2013). Use of Dicarboxylic Acids in Type 2 Diabetes. Br. J. Clin. Pharmacol..

[22] Goetzman E.S., Zhang B.B., Zhang Y., Bharathi S.S., Bons J., Rose J., Shah S., Solo K.J., Schmidt A.V., Richert A.C. (2024). Dietary Dicarboxylic Acids Provide a Non-Storable Alternative Fat Source That Protects Mice against Obesity. J. Clin. Investig..

[23] Zhang W., Zhang L., Yao H., Wang Y., Zhang X., Shang L., Chen X., Zeng J. (2023). Long-Chain Dicarboxylic Acids Play a Critical Role in Inducing Peroxisomal β-Oxidation and Hepatic Triacylglycerol Accumulation. J. Biol. Chem..

[24] Zhang X., Gao T., Deng S., Shang L., Chen X., Chen K., Li P., Cui X., Zeng J. (2021). Fasting Induces Hepatic Lipid Accumulation by Stimulating Peroxisomal Dicarboxylic Acid Oxidation. J. Biol. Chem..

[25] Pons R., Cavadini P., Baratta S., Invernizzi F., Lamantea E., Garavaglia B., Taroni F. (2000). Clinical and Molecular Heterogeneity in Very-Long-Chain Acyl-Coenzyme A Dehydrogenase Deficiency. Pediatr. Neurol..

[26] Tonsgard J.H., Mendelson S.A., Meredith S.C. (1988). Binding of Straight-Chain Saturated Dicarboxylic Acids to Albumin. J. Clin. Investig..

[27] Tonsgard J.H., Getz G.S. (1985). Effect of Reye’s Syndrome Serum on Isolated Chinchilla Liver Mitochondria. J. Clin. Investig..

[28] Olesen M.A., Villavicencio-Tejo F., Quintanilla R.A. (2022). The Use of Fibroblasts as a Valuable Strategy for Studying Mitochondrial Impairment in Neurological Disorders. Transl. Neurodegener..

[29] Barth P.G., Van den Bogert C., Bolhuis P.A., Scholte H.R., van Gennip A.H., Schutgens R.B., Ketel A.G. (1996). X-Linked Cardioskeletal Myopathy and Neutropenia (Barth Syndrome): Respiratory-Chain Abnormalities in Cultured Fibroblasts. J. Inherit. Metab. Dis..

[30] Djouadi F., Habarou F., Le Bachelier C., Ferdinandusse S., Schlemmer D., Benoist J.F., Boutron A., Andresen B.S., Visser G., de Lonlay P. (2016). Mitochondrial Trifunctional Protein Deficiency in Human Cultured Fibroblasts: Effects of Bezafibrate. J. Inherit. Metab. Dis..

[31] Wang D., Ho E.S., Cotticelli M.G., Xu P., Napierala J.S., Hauser L.A., Napierala M., Himes B.E., Wilson R.B., Lynch D.R. (2022). Skin Fibroblast Metabolomic Profiling Reveals That Lipid Dysfunction Predicts the Severity of Friedreich’s Ataxia. J. Lipid Res..

[32] Riazi R., Khairallah M., Cameron J.M., Pencharz P.B., Rosiers C.D., Robinson B.H. (2009). Probing Pyruvate Metabolism in Normal and Mutant Fibroblast Cell Lines Using 13C-Labeled Mass Isotopomer Analysis and Mass Spectrometry. Mol. Genet. Metab..

[33] Kølvraa S., Gregersen N., Christensen E., Hobolth N. (1982). In Vitro Fibroblast Studies in a Patient with C6-C10-Dicarboxylic Aciduria: Evidence for a Defect in General Acyl-CoA Dehydrogenase. Clin. Chim. Acta.

[34] Hannibal L., Theimer J., Wingert V., Klotz K., Bierschenk I., Nitschke R., Spiekerkoetter U., Grünert S.C. (2020). Metabolic Profiling in Human Fibroblasts Enables Subtype Clustering in Glycogen Storage Disease. Front. Endocrinol..

[35] Alatibi K.I., Hagenbuchner J., Wehbe Z., Karall D., Ausserlechner M.J., Vockley J., Spiekerkoetter U., Grünert S.C., Tucci S. (2021). Different Lipid Signature in Fibroblasts of Long-Chain Fatty Acid Oxidation Disorders. Cells.

[36] Junghans P., Görs S., Lang I.S., Steinhoff J., Hammon H.M., Metges C.C. (2010). A Simplified Mass Isotopomer Approach to Estimate Gluconeogenesis Rate in Vivo Using Deuterium Oxide. Rapid Commun. Mass Spectrom..

[37] House A., Fatica E., Shah R., Stergar J., Pearce R., Sandlers Y. (2019). A Protocol for Metabolic Characterization of Human Induced Pluripotent Stem Cell-Derived Cardiomyocytes (iPS-CM). MethodsX.

[38] Jin Z., Bian F., Tomcik K., Kelleher J.K., Zhang G.-F., Brunengraber H. (2015). Compartmentation of Metabolism of the C_12_-, C_9_-, and C_5_-*n*-Dicarboxylates in Rat Liver, Investigated by Mass Isotopomer Analysis. J. Biol. Chem..

[39] Bharathi S.S., Zhang Y., Gong Z., Muzumdar R., Goetzman E.S. (2020). Role of Mitochondrial Acyl-CoA Dehydrogenases in the Metabolism of Dicarboxylic Fatty Acids. Biochem. Biophys. Res. Commun..

[40] Liepinsh E., Makrecka-Kuka M., Volska K., Kuka J., Makarova E., Antone U., Sevostjanovs E., Vilskersts R., Strods A., Tars K. (2016). Long-Chain Acylcarnitines Determine Ischaemia/Reperfusion-Induced Damage in Heart Mitochondria. Biochem. J..

[41] Lefort B., Issa J., Blasco H., Labarthe F. (2022). Heart Failure Is Associated with Accumulation of Long Chain Acylcarnitines in Children Suffering from Cardiomyopathy. Arch. Cardiovasc. Dis. Suppl..

[42] Ruiz M., Labarthe F., Fortier A., Bouchard B., Legault J.T., Bolduc V., Rigal O., Chen J., Ducharme A., Crawford P.A. (2017). Circulating Acylcarnitine Profile in Human Heart Failure: A Surrogate of Fatty Acid Metabolic Dysregulation in Mitochondria and Beyond. Am. J. Physiol. Heart Circ. Physiol..

[43] Ahmad T., Kelly J.P., McGarrah R.W., Hellkamp A.S., Fiuzat M., Testani J.M., Wang T.S., Verma A., Samsky M.D., Donahue M.P. (2016). Prognostic Implications of Long-Chain Acylcarnitines in Heart Failure and Reversibility with Mechanical Circulatory Support. J. Am. Coll. Cardiol..

[44] Aitken-Buck H.M., Krause J., Zeller T., Jones P.P., Lamberts R.R. (2020). Long-Chain Acylcarnitines and Cardiac Excitation-Contraction Coupling: Links to Arrhythmias. Front. Physiol..

[45] Liepinsh E., Kuka J., Vilks K., Svalbe B., Stelfa G., Vilskersts R., Sevostjanovs E., Goldins N.R., Groma V., Grinberga S. (2021). Low Cardiac Content of Long-Chain Acylcarnitines in TMLHE Knockout Mice Prevents Ischaemia-Reperfusion-Induced Mitochondrial and Cardiac Damage. Free Radic. Biol. Med..

[46] Kuka J., Makrecka-Kuka M., Cirule H., Grinberga S., Sevostjanovs E., Dambrova M., Liepinsh E. (2017). Decrease in Long-Chain Acylcarnitine Tissue Content Determines the Duration of and Correlates with the Cardioprotective Effect of Methyl-GBB. Basic Clin. Pharmacol. Toxicol..

[47] Radzikh I., Fatica E., Kodger J., Shah R., Pearce R., Sandlers Y.I. (2021). Metabolic Outcomes of Anaplerotic Dodecanedioic Acid Supplementation in Very Long Chain Acyl-CoA Dehydrogenase (VLCAD) Deficient Fibroblasts. Metabolites.

[48] Makievskaya C.I., Popkov V.A., Andrianova N.V., Liao X., Zorov D.B., Plotnikov E.Y. (2023). Ketogenic Diet and Ketone Bodies against Ischemic Injury: Targets, Mechanisms, and Therapeutic Potential. Int. J. Mol. Sci..

[49] Ventura F.V., Ruiter J.P.N., IJlst L., de Almeida I.T., Wanders R.J.A. (1998). Lactic Acidosis in Long-Chain Fatty Acid Beta-Oxidation Disorders. J. Inherit. Metab. Dis..

[50] Koenig M.K. (2008). Presentation and Diagnosis of Mitochondrial Disorders in Children. Pediatr. Neurol..

[51] Cade W.T., Bohnert K.L., Peterson L.R., Patterson B.W., Bittel A.J., Okunade A.L., de las Fuentes L., Steger-May K., Bashir A., Schweitzer G.G. (2019). Blunted Fat Oxidation upon Submaximal Exercise Is Partially Compensated by Enhanced Glucose Metabolism in Children, Adolescents, and Young Adults with Barth Syndrome. J. Inherit. Metab. Dis..

[52] Jones P.M., Bennett M.J. (2017). Disorders of Mitochondrial Fatty Acid β-Oxidation. Biomarkers in Inborn Errors of Metabolism.

[53] Fillmore N., Levasseur J.L., Fukushima A., Wagg C.S., Wang W., Dyck J.R.B., Lopaschuk G.D. (2018). Uncoupling of Glycolysis from Glucose Oxidation Accompanies the Development of Heart Failure with Preserved Ejection Fraction. Mol. Med..

[54] Wu P., Zhu T., Huang Y., Fang Z., Luo F. (2023). Current Understanding of the Contribution of Lactate to the Cardiovascular System and Its Therapeutic Relevance. Front. Endocrinol..

[55] Lopaschuk G.D., Karwi Q.G., Tian R., Wende A.R., Abel E.D. (2021). Cardiac Energy Metabolism in Heart Failure. Circ. Res..

[56] Amaral A.U., Cecatto C., Da Silva J.C., Wajner A., Wajner M. (2017). Mechanistic Bases of Neurotoxicity Provoked by Fatty Acids Accumulating in MCAD and LCHAD Deficiencies. J. Inborn Errors Metab. Screen..

[57] Tonin A.M., Amaral A.U., Busanello E.N.B., Grings M., Castilho R.F., Wajner M. (2013). Long-Chain 3-Hydroxy Fatty Acids Accumulating in Long-Chain 3-Hydroxyacyl-CoA Dehydrogenase and Mitochondrial Trifunctional Protein Deficiencies Uncouple Oxidative Phosphorylation in Heart Mitochondria. J. Bioenerg. Biomembr..

[58] Fillmore N., Lopaschuk G.D. (2013). Targeting Mitochondrial Oxidative Metabolism as an Approach to Treat Heart Failure. Biochim. Biophys. Acta (BBA) Mol. Cell Res..

[59] Nassiri S., Van De Bovenkamp A.A., Remmelzwaal S., Sorea O., De Man F., Handoko M.L. (2024). Effects of Trimetazidine on Heart Failure with Reduced Ejection Fraction and Associated Clinical Outcomes: A Systematic Review and Meta-Analysis. Open Heart.

[60] Fragasso G., Spoladore R., Cuko A., Palloshi A. (2007). Modulation of Fatty Acids Oxidation in Heart Failure by Selective Pharmacological Inhibition of 3-Ketoacyl Coenzyme-A Thiolase. Curr. Clin. Pharmacol..

[61] O’Connor R.S., Guo L., Ghassemi S., Snyder N.W., Worth A.J., Weng L., Kam Y., Philipson B., Trefely S., Nunez-Cruz S. (2018). The CPT1a Inhibitor, Etomoxir Induces Severe Oxidative Stress at Commonly Used Concentrations. Sci. Rep..

[62] Cox P.J., Kirk T., Ashmore T., Willerton K., Evans R., Smith A., Murray A.J., Stubbs B., West J., McLure S.W. (2016). Nutritional Ketosis Alters Fuel Preference and Thereby Endurance Performance in Athletes. Cell Metab..

[63] Bleeker J.C., Visser G., Clarke K., Ferdinandusse S., de Haan F.H., Houtkooper R.H., IJlst L., Kok I.L., Langeveld M., van der Pol W.L. (2020). Nutritional Ketosis Improves Exercise Metabolism in Patients with Very Long-Chain Acyl-CoA Dehydrogenase Deficiency. J. Inherit. Metab. Dis..

[64] Hashim S.A., Vanitallie T.B., Luke’s- S., Hospital R. (2014). Ketone Body Therapy: From the Ketogenic Diet to the Oral Administration of Ketone Ester. J. Lipid Res..

[65] Veech R.L. (2004). The Therapeutic Implications of Ketone Bodies: The Effects of Ketone Bodies in Pathological Conditions: Ketosis, Ketogenic Diet, Redox States, Insulin Resistance, and Mitochondrial Metabolism. Prostagland. Leukot. Essent. Fat. Acids.

[66] Ho K.L., Karwi Q.G., Wang F., Wagg C., Zhang L., Panidarapu S., Chen B., Pherwani S., Greenwell A.A., Oudit G.Y. (2024). The Ketogenic Diet Does Not Improve Cardiac Function and Blunts Glucose Oxidation in Ischaemic Heart Failure. Cardiovasc. Res..

[67] Greco C.M., Nisoli E. (2024). The Ketogenic Diet Is Unable to Improve Cardiac Function in Ischaemic Heart Failure: An Unexpected Result?. Cardiovasc. Res..

[68] Ho K.L., Karwi Q.G., Wagg C., Zhang L., Vo K., Altamimi T., Uddin G.M., Ussher J.R., Lopaschuk G.D. (2020). Ketones Can Become the Major Fuel Source for the Heart but Do Not Increase Cardiac Efficiency. Cardiovasc. Res..

[69] Karwi Q.G., Lopaschuk G.D. (2022). CrossTalk Proposal: Ketone Bodies Are an Important Metabolic Fuel for the Heart. J. Physiol..

[70] Ho K.L., Zhang L., Wagg C., Al Batran R., Gopal K., Levasseur J., Leone T., Dyck J.R.B., Ussher J.R., Muoio D.M. (2019). Increased Ketone Body Oxidation Provides Additional Energy for the Failing Heart without Improving Cardiac Efficiency. Cardiovasc. Res..

