# Supplementary material for: Dodecanedioic Acid: Alternative Carbon Substrate or Toxic Metabolite?
Source: Biomolecules. 2025 Dec 30;16(1):57. doi: 10.3390/biom16010057 (PMC12839351; doi:10.3390/biom16010057)
Supplement: Supplementary file 1 [file biomolecules-16-00057-s001.zip › Table S1_FINAL.pdf]

Name	Retention Time	M0	M1	M2	M3	M4	M5	M6	M7	M8	M9	M10	M11	M12
**Citrate**	16.78	459	460	461	462	463	464	465						
**Ketoglutarate**	14.28	345	346	347	348	349	350							
**Succinate**	11.59	289	290	291	292	293								
**Fumarate**	11.78	287	288	289	290	291								
**Malate**	13.95	419	420	421	422	423								
**DODA**	16.54	401	402	403	404	405	406	407	408	409	410	411	412	413

Table S1: Retention times and target ions (GCMS). All metabolites TBDMS derivatives
