# Supplementary material for: Dodecanedioic Acid: Alternative Carbon Substrate or Toxic Metabolite?
Source: Biomolecules. 2025 Dec 30;16(1):57. doi: 10.3390/biom16010057 (PMC12839351; doi:10.3390/biom16010057)
Supplement: Supplementary file 1 [file biomolecules-16-00057-s001.zip › Table S2_FINAL.pdf]

Table S2: Variable importance in projection (VIP) scores for metabolites contributing to variation in metabolic profiles of (-) DODA vs. (+) DODA cells

	V1	V2
Dodecanedioic Acid	2.125610272	0.187580968
C16 H32 O3	2.09159697	0.288876348
PS(22:0/18:3(6Z,9Z,12Z))	2.081738632	0.155160402
C12 H22 O3	2.067490297	0.351287301
Dodecanoylcarnitine	2.063311682	0.342862746
L-Glutamic acid	2.009812382	0.478571967
Palmitoylcarnitine	2.004847758	0.474439342
C10 H20 O3	1.986733167	0.132823562
PS(20:4(5Z,8Z,11Z,14Z)/18:0)	1.973596991	0.220755005
C18 H36 O3	1.969695035	0.362746728
C14 H26 O3	1.943877118	0.253714291
Citric Acid	1.909535714	0.639966797
Acetoacetic acid	1.891469773	0.377547713
Taurine	1.891100347	0.636556482
C9 H15 N4 O8 P	1.88057377	0.447694188
Tetradecenoyl-L-carnitine	1.830045442	0.02701008
Capric acid	1.810990777	0.27358017
C43 H80 O10	1.769928512	0.499570186
GSH / Glutathione	1.7674081	0.481526569
UDP-N-acetyl-D-mannosamine	1.761832806	0.518420753
C5 H10 O3	1.738530107	0.731165393
Succinic acid	1.729152557	0.851696325
C14 H22 O	1.728265664	0.005192808
C18 H34 O3	1.719917063	0.237605489
PS(19:0/19:0)	1.694101169	0.445089386
Malic acid	1.608438508	0.944424934
C8 H12 O4.1	1.599893071	0.516966413
PS(20:0/18:1(9Z))	1.5811481	0.218988699
PS(22:1(11Z)/20:2(11Z,14Z))	1.563385025	0.495767072
PI(20:4(5Z,8Z,11Z,14Z)/0:0)	1.558116921	0.758258294
Lauric acid	1.555666438	0.463123199
Lucidenic acid M	1.53553858	0.198334909
C12 H20 O2	1.496320625	0.890205094
C16 H30 O2	1.452502846	0.385996281
C6 H12 O3	1.426367252	0.447864431
C12 H21 N O8 S	1.404373237	0.922240164
PI(18:0/0:0)	1.361349029	0.225007333

C10 H11 N O3	1.352407091	0.93799208
C14 H22 O4	1.343255036	0.680935126
LysoPE(22:5(7Z,10Z,13Z,16Z,19Z)/0:0)	1.341241534	0.927355239
C13 H10 S	1.338667765	0.824580141
C8 H12 O4	1.333646422	0.948352135
C11 H14 N4 O4 S	1.319439878	0.531157906
L-Proline	1.308172121	1.009689395
C6 H13 O8 P	1.305670645	0.903083734
Creatinine	1.281250695	1.146032526
L-Glutamine	1.269533587	0.333520084
C9 H18 O8	1.266837347	0.969221812
C6 H5 N O3	1.262902215	0.959293034
Butyric acid	1.244209012	0.9418184
PI(18:1(9Z)/0:0)	1.242041928	0.414804784
C5 H12 O5	1.227857538	1.184832048
C7 H12 O3	1.224055657	0.92134839
C16 H21 N O11	1.216035712	0.979781811
PI(18:0/20:4(5Z,8Z,11Z,14Z))	1.197473487	0.873969716
C6 H9 N O4	1.195559877	1.035200972
C25 H44 N O7 P	1.188973061	1.0533797
alpha-Ketoglutaric acid	1.164451304	0.61508243
Fumaric acid	1.163257943	1.197236579
C17 H26 O4	1.156457218	0.484281152
C7 H13 O6 P	1.152297417	1.215645943
L-Serine	1.15053635	1.211349178
L-Homocystine	1.113176242	1.071692813
C5 H11 O7 P	1.112780479	0.918254365
C14 H21 N O3	1.107309568	1.066332375
C25 H42 O6	1.08365634	0.428128874
Adenosine 5'-triphosphate (ATP)	1.07081698	1.110178152
C10 H14 N6 O4	1.067927205	1.263584121
C28 H12 O16	1.060979188	0.43028133
C8 H14 N2 O5	1.057807808	1.17446065
D-Fructose 6-phosphate	1.050044618	1.291692499
C12 H26 O4 S	1.049531363	0.551283926
C20 H16 N6 O2 S	1.039979964	1.208015711
C12 H23 N2 O9 P S	1.019343123	1.257651683
C9 H16 O5 S	0.999281873	1.032199564
C13 H26 O6	0.998063659	1.070301146
C5 H13 O7 P	0.969655211	1.167343837
Decanoyl acetaldehyde	0.960513971	1.212730485
C3 H7 N O4 S	0.958844378	1.09181115

C10 H15 N2 O7	0.946402127	1.284706988
C7 H10 O6	0.946053897	1.038170017
C12 H8 O4	0.926086207	0.944361683
LysoPE(0:0/16:0)	0.924859861	0.897058635
C5 H12 N O5 P	0.924857397	1.282621287
C H4 O4 S	0.917047687	1.174133646
C4 H7 N O3	0.91456495	1.098507465
D-(-)-?-Hydroxybutyric acid	0.896687998	1.22821571
Lactic acid	0.89338386	0.787927313
L-Threonine	0.889115162	1.009691201
C9 H16 O3	0.888702755	0.068162167
C12 H18 N2 O7	0.886274326	0.811409071
C8 H11 N O3.1	0.868987996	0.661659387
L-Lyxonate	0.868188209	1.289074917
C17 H18 O4	0.859821495	0.905112531
L-Phenylalanine	0.856287344	1.312208337
C9 H18 O2	0.854498795	0.673088848
C7 H15 O9 P	0.849683224	1.22126888
C23 H30 O12	0.84833879	1.175086038
L-Aspartic Acid	0.842402045	1.300056108
C6 H11 N O5	0.835228114	1.019281537
Glycine	0.805487447	0.856072649
C17 H34 O2	0.803114316	1.211074985
L-Tyrosine	0.79434452	1.202510749
C5 H7 N O S	0.792449674	0.613292218
C10 H11 N O3.1	0.792446657	1.363101103
Caprylic acid	0.783921283	1.006151963
C8 H9 N O4	0.778180429	1.370213946
C10 H8 O3 S	0.776813284	1.319597869
C8 H15 N O4	0.775991966	1.370039319
C6 H8 O5	0.774182501	0.993744704
C8 H6 O4	0.761025241	1.263704032
L-Galactose	0.745807908	1.010725665
C22 H28 O12	0.741060821	0.557107941
C12 H19 N5 O4 S	0.740529861	1.339800051
C7 H13 N O3	0.73762146	1.192214533
PEP / Phosphoenolpyruvic acid	0.737035743	1.340706111
C18 H30 O3 S	0.683790422	1.180482452
C18 H34 O4	0.678700761	0.656734076
Folic acid	0.67506436	1.309147516
C7 H6 O3	0.674683801	0.507360444
C25 H44 N O7 P.1	0.663310127	0.09146149

C9 H16 O9	0.661391955	1.364864754
C6 H4 N2 O5	0.659535824	1.30542372
L-Arginine	0.652932824	1.419199032
C6 H8 O4.1	0.642003187	0.7398454
C12 H18 O13	0.638634383	1.273660371
C17 H20 N2 O6 S	0.632182208	1.313028586
C22 H36 O2	0.623848144	0.889644159
Arachidyl carnitine	0.581149491	0.775034838
C3 H4 O2	0.567069177	0.709922186
C8 H14 O8	0.556490491	1.315693153
C11 H22 O2	0.553309219	0.60169067
C17 H32 O2	0.546907749	1.374269334
PI(20:3(8Z,11Z,14Z)/0:0)	0.545754462	1.18536262
C45 H76 N O7 P	0.545454848	0.700018412
C4 H6 O7 S	0.542766259	1.248408551
C14 H11 N O4	0.541917135	1.198580138
C9 H16 O3.1	0.537318831	0.26549438
PS(22:0/0:0)	0.535932334	0.676412075
C6 H11 N O5.1	0.531616634	1.3266751
C4 H6 N4 O3	0.528214014	1.356325493
C12 H15 N O4	0.520829338	1.294353161
C12 H18 O2	0.520728596	1.11951014
PS(22:0/20:4(5Z,8Z,11Z,14Z))	0.515929673	0.328745611
C9 H19 O11 P	0.512343036	1.396294003
C11 H21 N O7 S	0.498824627	1.037930442
C12 H21 N O9	0.498234545	1.211710413
C15 H12 O7	0.483225912	0.873794712
C13 H10 N2 O5 S	0.474720968	0.982779472
C8 H11 N O3	0.463606365	1.412783892
C9 H18 O3	0.460613934	0.479468155
C4 H8 S Se	0.458125313	0.20882763
C20 H22 N2 O7 S	0.454420002	0.961153654
Uridine 5'-triphosphate (UTP)	0.449034816	1.415330822
C5 H8 O2	0.442387537	1.184720365
C6 H8 O4	0.428392564	0.604754529
His Gly Cys	0.425128134	1.422031272
PS(22:2(13Z,16Z)/18:4(6Z,9Z,12Z,15Z))	0.420839794	0.5571473
C13 H16 O5	0.420269144	1.136838308
L-Methionine	0.412891985	1.460546367
ADP / Adenosine 5'-diphosphate	0.40428374	1.401521363
Demethylphosphinothricin	0.40235626	1.424218401
C20 H32 O2	0.399669838	0.668901954

C5 H10 O4 S2	0.399304504	0.928803069
C13 H10 O6	0.387544239	1.250886377
C11 H12 N2 O3 S	0.365462331	1.390704525
C13 H19 N O4	0.33997479	1.400540043
C9 H17 N O3	0.332403958	1.238643145
C11 H16 N5 O5	0.310396215	0.686501031
D-4'-Phosphopantothenate	0.301862382	1.399807358
L-Cartinine	0.301666232	0.843448893
Pro Glu His	0.293308421	1.375328824
Lys Gln Arg	0.292045847	1.400030547
C10 H12 N4 O5	0.288696983	1.063121269
PS(18:1(9Z)/0:0)	0.284411829	1.116508137
C13 H10 N2 O5 S.1	0.280383767	1.068842289
L-Lysine	0.279727571	1.445334195
C9 H14 N2 O6	0.268451409	1.429734152
C5 H4 O3	0.260929629	1.456176091
C14 H26 O2	0.256126757	0.73311008
PS(22:2(13Z,16Z)/18:3(6Z,9Z,12Z))	0.2494821	0.506049047
C6 H10 N2 O5	0.249193207	1.414375557
C4 H9 N3 O2	0.243196846	0.895258193
C4 H8 O2 S	0.233109323	0.285625776
C7 H8 O S.1	0.226767338	1.136679295
UDPG / Uridine 5'-diphosphoglucose	0.204593099	1.438691086
C6 H9 N2 O5 P	0.202915236	0.985758232
C16 H20 N2 O12 S3	0.199976086	1.435105407
C9 H10 O4	0.190525586	1.375228513
C18 H34 N4 O6	0.190394505	1.262505711
C5 H6 O3	0.172338636	0.54301259
C11 H14 O3	0.168885188	0.676141579
C12 H20 O4	0.165038949	0.289947403
C11 H17 N O8	0.162041861	0.938832693
C22 H32 O2	0.156309359	0.600599474
C8 H9 N O3	0.155241768	1.087331257
C4 H7 O8 P	0.149207463	1.371174776
C6 H8 O6	0.141340829	1.304098936
L-Alanine	0.139287539	1.184006156
L-Cysteine	0.137781691	0.210574683
Cysteinyl-Cysteine	0.119917814	0.875390898
C6 H6 N2 O2	0.11734339	0.747847524
C18 H30 O3 S.1	0.116033732	0.863718171
C10 H18 O5 S	0.113356819	0.530594485
PHOSPHORYLCHOLINE	0.112915692	1.465271497

Pyruvic acid	0.112224539	1.480982896
C14 H18 O9	0.111123073	1.154606449
C13 H22 O4	0.11010922	0.804569175
L-Isoleucine	0.101601139	1.459672249
C15 H23 N5 O7	0.096474295	1.488049333
C7 H8 O S	0.095328229	1.160809525
D-fructose 1,6-bisphosphate	0.083389182	1.461110745
L-Asparagine	0.083210166	1.117908545
L-Fuculose 1-phosphate	0.077597958	0.780563721
C8 H14 O3	0.074481921	0.839881838
L-Leucine	0.058611256	0.990335976
PS(18:1(9Z)/22:0)	0.026745038	1.091519584
PS(22:0/14:1(9Z))	0.000823369	1.394199535
