# Supplementary material for: Dodecanedioic Acid: Alternative Carbon Substrate or Toxic Metabolite?
Source: Biomolecules. 2025 Dec 30;16(1):57. doi: 10.3390/biom16010057 (PMC12839351; doi:10.3390/biom16010057)
Supplement: Supplementary file 1 [file biomolecules-16-00057-s001.zip › Table S3_LIPIDS_FINAL.pdf]

Lipid Type	Lipid Class	- DODA (%)	+ DODA(%)
Glycerophospholipids	PE	24.15	23.10
	EtherPE	13.21	12.27
	PI	11.32	11.91
	PG	11.32	9.03
	PC	3.4	5.78
	OxPI	2.26	2.89
	PS	1.89	2.17
	EtherPC	1.89	1.81
	LPI	1.51	1.81
	OxPE	0.75	1.81
	LPE	0.75	1.08
Sphingolipids	Cer	13.21	11.91
	SM	4.15	4.69
	GM	4.53	3.97
	HexCer	2.64	2.17
Fatty Acids	FA	3.02	2.53

Table 2: Identified lipids classes by LC-QTOF in negative mode

PE: Phosphatidylethanolamines; **EtherPE:** Ether-linked phosphatidylethanolamines; **PI:** Phosphatidylinositols; **PG:** Phosphatidylglycerols; **PC:** Phosphatidylcholines; **OxPI:** Oxidized phosphatidylinositols; **PS:** Phosphatidylserines; **EtherPC:** Ether-linked phosphatidylcholines; **LPI:** Lysophosphatidylinositols; **OxPE:** Oxidized phosphatidylethanolamines; **LPE:** Lysophosphatidylethanolamines; **Cer:** Ceramides; **SM:** Sphingomyelins; **GM:** Gangliosides; **HexCer:** Hexosylceramides; **FA:** Fatty Acids; **LPC:** Lysophosphatidylcholines; **TG:** Triglycerides; **DG:** Diglycerides; **CE:** Cholesterol Esters

Lipid Type	Lipid Type	- DODA (%)	+ DODA (%)
Glycerophospholipids	PC	34.84	33.33
	LPC	7.38	6.14
	PE	5.74	5.70
	LPE	2.87	3.51
	PI	0.82	0.88
Glycerols	TG	19.26	20.61
	DG	2.46	1.32
Sphingolipids	SM	11.48	13.60
	Cer	8.20	7.89
Fatty acids	FA	5.33	5.26
Sterols	CE	1.64	1.75

Table 1a: Identified lipids classes by LC-QTOF in positive mode

PE: Phosphatidylethanolamines; **EtherPE:** Ether-linked phosphatidylethanolamines; **PI:** Phosphatidylinositols; **PG:** Phosphatidylglycerols; **PC:** Phosphatidylcholines; **OxPI:** Oxidized phosphatidylinositols; **PS:** Phosphatidylserines; **EtherPC:** Ether-linked phosphatidylcholines; **LPI:** Lysophosphatidylinositols; **OxPE:** Oxidized phosphatidylethanolamines; **LPE:** Lysophosphatidylethanolamines; **Cer:** Ceramides; **SM:** Sphingomyelins; **GM:** Gangliosides; **HexCer:** Hexosylceramides; **FA:** Fatty Acids; **LPC:** Lysophosphatidylcholines; **TG:** Triglycerides; **DG:** Diglycerides; **CE:** Cholesterol Esters
