# Supplementary figures and images for: Dodecanedioic Acid: Alternative Carbon Substrate or Toxic Metabolite?
Source: Biomolecules. 2025 Dec 30;16(1):57. doi: 10.3390/biom16010057 (PMC12839351; doi:10.3390/biom16010057)

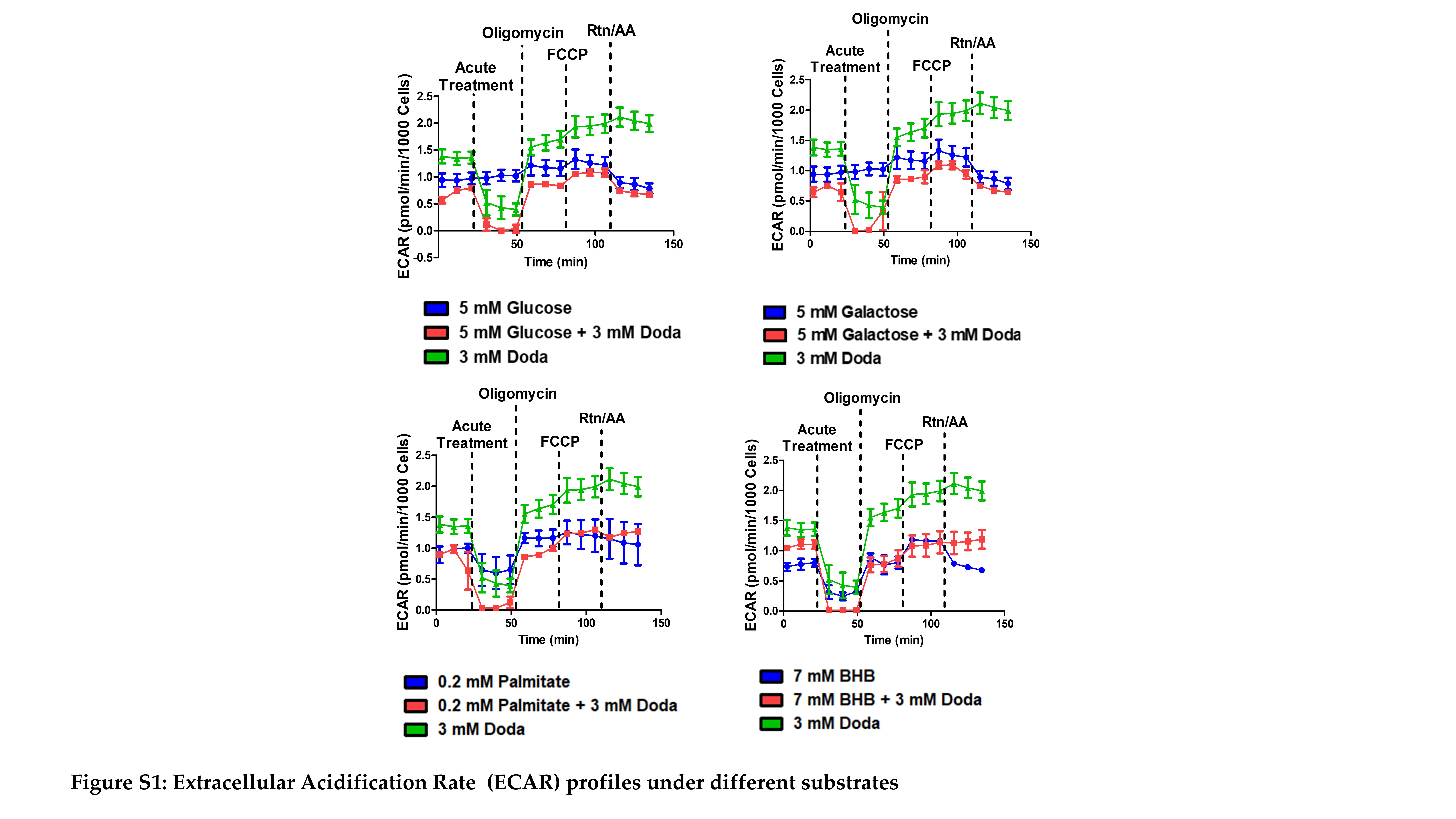

Supplement: Supplementary file 1 [file biomolecules-16-00057-s001.zip › Fig_1S_REVISED.jpg]
